# Brain site-specific proteome changes in aging-related dementia

**DOI:** 10.1038/emm.2013.76

**Published:** 2013-09-06

**Authors:** Arulmani Manavalan, Manisha Mishra, Lin Feng, Siu Kwan Sze, Hiroyasu Akatsu, Klaus Heese

**Affiliations:** 1 School of Biological Sciences, Nanyang Technological University, Singapore, Singapore; 2 Department of Obstetrics and Gynecology, University of California Irvine, Irvine, CA, USA; 3 Choju Medical Institute, Fukushimura Hospital,Toyohashi, Aichi, Japan; 4 Graduate School of Biomedical Science and Engineering, Hanyang University, Seoul, Republic of Korea

**Keywords:** aging, Alzheimer's disease, proteasome, proteomics, ubiquitin

## Abstract

This study is aimed at gaining insights into the brain site-specific proteomic senescence signature while comparing physiologically aged brains with aging-related dementia brains (for example, Alzheimer's disease (AD)). Our study of proteomic differences within the hippocampus (Hp), parietal cortex (pCx) and cerebellum (Cb) could provide conceptual insights into the molecular mechanisms involved in aging-related neurodegeneration. Using an isobaric tag for relative and absolute quantitation (iTRAQ)-based two-dimensional liquid chromatography coupled with tandem mass spectrometry (2D-LC-MS/MS) brain site-specific proteomic strategy, we identified 950 proteins in the Hp, pCx and Cb of AD brains. Of these proteins, 31 were significantly altered. Most of the differentially regulated proteins are involved in molecular transport, nervous system development, synaptic plasticity and apoptosis. Particularly, proteins such as Gelsolin (GSN), Tenascin-R (TNR) and AHNAK could potentially act as novel biomarkers of aging-related neurodegeneration. Importantly, our Ingenuity Pathway Analysis (IPA)-based network analysis further revealed ubiquitin C (UBC) as a pivotal protein to interact with diverse AD-associated pathophysiological molecular factors and suggests the reduced ubiquitin proteasome degradation system (UPS) as one of the causative factors of AD.

## Introduction

Understanding the unique alterations in the brain proteomic signature induced by cellular senescence as a result of normal and pathological aging is essential for identification of a specific cure for various neurodegenerative disorders. Alzheimer's disease (AD) is a progressive, aging-related neurodegenerative disorder of the central nervous system and is the most common cause of dementia in the elderly worldwide. It is characterized by impaired memory and the deterioration of higher cognitive functions.^1, 2^ The main pathological characteristics of AD are accumulations of senile plaques that comprise amyloid-β (Aβ) peptides produced from the β-amyloid precursor protein (APP) following sequential processing by β- and γ-secretases.^3, 4, 5, 6^ The second major pathological hallmark of AD is neurofibrillary tangles (NFTs) caused by intracellular accumulations of the hyperphosphorylated microtubule-associated protein tau (MAPT).^7, 8, 9^ Neurodegeneration in AD progresses sequentially, starting first in predisposed induction sites from the entorhinal cortex in the medial temporal lobe and then gradually spreading to the entire hippocampus (Hp) and the limbic system, before advancing in topographically predictable sequences and ultimately expanding to the temporal association cortex. Although the occipital lobe cortex retains nearly normal function, frequently even in terminal-stage patients, the temporal lobe cortex, in contrast, as one of the most fragile parts of the brain, is extremely vulnerable to neuronal death.^10, 11, 12, 13^ Thus, it might be possible to identify those biomarkers that cause aging-related AD in the temporal lobe. Biomarkers that are capable of preventing neurodegenerative processes within the occipital lobe at early stages of AD may also be identified.^14, 15^ Because the prevailing assumption is that the cerebellar proteome of the central nervous system area is relatively unaffected by AD, this is a less investigated region and often neglected in AD studies. However, it must be noted that several studies indicated that in AD the cerebellum (Cb) also shows some signs of morphological and metabolic dysfunctions.^16^ A number of pathological changes, such as widespread deposits of diffuse amyloid, ubiquitin-immunoreactive dystrophic neurites and increased microglia, have been revealed in the AD-affected Cb by immunocytochemical studies. However, tau-immunoreactive NFTs have not been seen. Although the observed changes may be merely epiphenomenal to the pathological processes occurring in the AD neocortex and Hp, the morphological and immunocytochemical differences between cerebral and cerebellar cortices of AD patients may nonetheless give insights into the molecular factors involved in the development of the neuropathological lesions of the AD brain.^16^ Consequently, more attention has also been given to this brain area in recent years, though with little focus at the molecular level.^17, 18, 19, 20, 21, 22, 23, 24^

Thus far, proteomic analyses have been considered worldwide with greater emphasis on improving the diagnosis of early stages of AD by identifying novel specific biomarkers from the cerebrospinal fluid or plasma to complement the existing diagnostic methods that are based on autopsy or neuroimaging techniques. Isobaric tags for relative and absolute quantitation (iTRAQ) is a widely accepted approach for quantitative proteomics.^25^ Accordingly, in this study, we applied the two-dimensional (2D) liquid chromatography coupled with tandem mass spectrometry-based iTRAQ (2D-LC-MS/MS-iTRAQ) technique for quantitative profiling of aging-related brain site-specific proteome changes. We sought to investigate the mechanisms of senescence and pathological aging-related neurodegeneration at the proteomic level in different areas of the brain, including the Hp, the parietal cortex (pCx) and the Cb, by comprehensively identifying the proteins with altered expression levels in response to aging and aging-related dementia (for example, AD). Subsequently, we exploited publically accessible bioinformatic databases to infer the functional role and networks linked to the proteins that we found differentially expressed.

Our data also reveal protein pathway modulations acquired by the Cb to counteract neurodegeneration and thus could eventually become a lucrative target for the identification of plausible therapeutic drugs.

## Materials and methods

### Reagents

Unless indicated, all reagents used for biochemical methods were purchased from Sigma-Aldrich (St Louis, MO, USA). Materials and reagents for sodium dodecyl sulfate-polyacrylamide gel electrophoresis were from Bio-Rad (Bio-Rad Laboratories, Hercules, CA, USA). The iTRAQ reagent eight-plex kit was bought commercially (Applied Biosystems, Foster City, CA, USA).

### Human subjects

Brain tissues were obtained from the brain bank of the Choju Medical Institute of the Fukushimura hospital (Toyohashi, Aichi, Japan), and the protocols utilized were approved by the local ethics committee of the Fukushimura hospital. The scientific use of human material was conducted in accordance with the Declaration of Helsinki, and informed consent was obtained from the guardians of the patients. Brain tissues were weighed at the time of autopsy, snap frozen with liquid nitrogen and stored at –80 °C. Patients with sporadic AD (early stage, low incidence, Table 1) received a pathological diagnosis according to the criteria of the Consortium to Establish a Registry for Alzheimer's Disease (CERAD) and the Braak stage^13, 26^ as described previously.^27, 28^ Controls were elderly patients who were age matched but without significant neurological disorders. Patients were also cognitively evaluated by neuropsychological tests using the mini-mental state examination (MMSE) and Hasegawa's dementia scale (HDS, or the HDS revised version (HDS-R)), which is commonly utilized in Japan.^14, 15, 27, 28, 29^

### Autopsy, neuropathological diagnostic criteria and immunohistochemistry

Brains were removed at the time of autopsy, weighed, cut mid-sagittally and examined for vascular and other macroscopically detectable lesions. Specimens for diagnostic examination were obtained from the hemisphere showing abnormal findings based on computed tomography scanning or from the left hemisphere when no difference was observed between the left and the right hemispheres. Specimens were fixed in 4% paraformaldehyde (Sigma, Tokyo, Japan) as a hemisphere block. Samples used for diagnostic purposes were taken from the frontal, temporal, parietal and occipital lobes; hippocampal formation; amygdala; basal ganglia; thalamus; and midbrain, including the substantia nigra, pons, medulla and the Cb. The specimens were embedded in paraffin and processed into 5-mm sections for conventional histological and immunohistochemical examination. For standard tissue characterization, specimens were stained using hematoxylin–eosin (to display morphology) and Klüver–Barerra staining methods. Methenamine silver and modified Gallyas–Braak staining were used to detect senile plaques, cerebral amyloid angiopathy and NFTs. Further characterization by immunohistochemistry was performed with specific antibodies against ubiquitin, α-synuclein, Aβ (1–40/42), and MAPT (IBL, Fujioka, Japan). Immunostaining methods were applied using the antibodies in a dilution of 1:1000 and a standard Avidin–Biotin Complex (ABC) method using a VectaStain Elite ABC kit (Vector Laboratories, Burlingame, CA, USA) and the protocols provided by the supplier. After being extensively washed, immunoreactive products were detected with DAKO Envision+ (Dako, Kyoto, Japan) and visualized after the addition of diaminobenzidine as the chromogen.^14, 15, 27, 28, 29^

### Human brain tissue protein extraction from Hp, pCx and Cb

Briefly, brain tissues from Hp, pCx and Cb (Table 1) were excised, snap-frozen in liquid nitrogen and then powdered using a mortar and pestle. Upon addition of lysis buffer (2% sodium dodecyl sulfate, 0.5 ℳ triethyl ammonium bicarbonate buffer (TEAB), 1 Complete protease inhibitor cocktail tablet (Roche, Mannheim, Germany) and 1 PhosSTOP phosphatase inhibitor cocktail tablet (Roche)), the samples were vortexed for 1 min and incubated on ice for an additional 45 min before homogenization (sonication parameters: amplitude, 23% pulse: 5 s/5 s for 5 min) using a Vibra Cell high intensity ultrasonic processor (Jencons Scientific, Leighton Buzzard, Bedfordshire, UK). After centrifugation (20 000 × *g*/4 °C/30 min), supernatant was collected and stored at −80 °C until further use. The protein was quantified by a ‘2-D Quant' kit (Amersham, Piscataway, NJ, USA) according to the manufacturer's protocol.^14, 29^

### iTRAQ protocol

A detailed description of the 2D-LC-MS/MS-iTRAQ procedures,^30, 31^ including postproteomic data verification by sodium dodecyl sulfate-polyacrylamide gel electrophoresis and western blot analyses (Supplementary Figures S1–S3),^32, 33, 34^ can be found in the Supplementary document.

#### Experimental design

**Figure 1:**
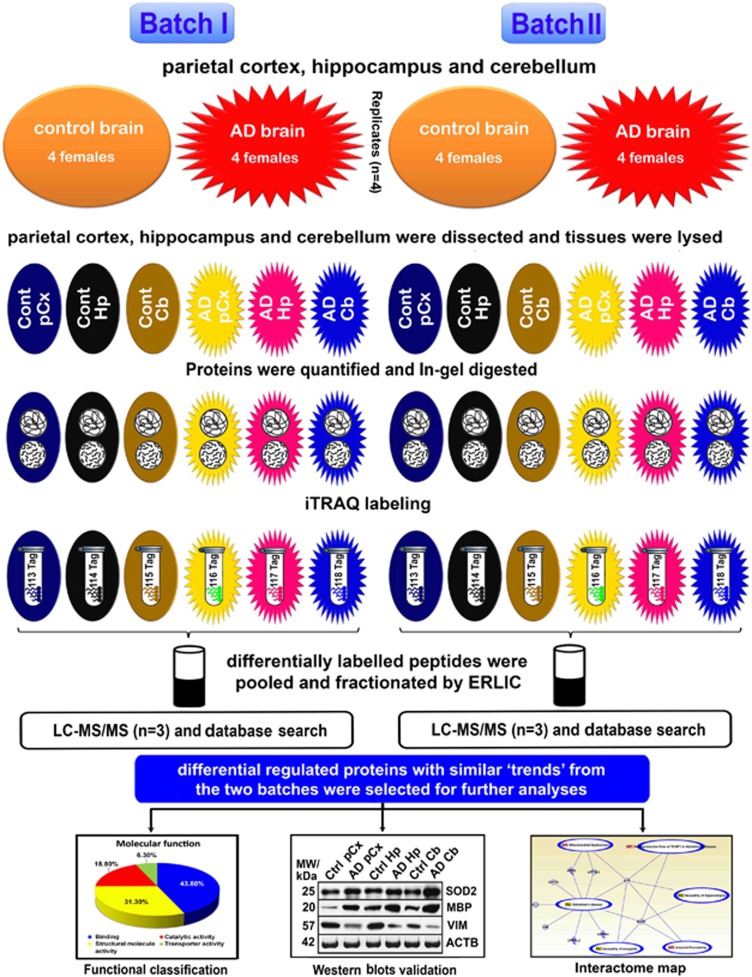
Schematic representation of the experimental design showing biological and technical replicates. Brain tissues (hippocampus (Hp), parietal cortex (pCx) and cerebellum (Cb)) were isolated from four Alzheimer's disease (AD) and four age-matched non-demented control subjects. The quantitative proteomics analyses of AD and control brains were performed by labeling with multi eight-plex isobaric tag for relative and absolute quantitation (iTRAQ) reagents (113–118) (followed by electrostatic repulsion-hydrophilic interaction chromatography (ERLIC)-based fractionation, and liquid chromatography coupled with tandem mass spectrometry (LC-MS/MS)-based multidimensional protein identification technology (2D-LC-MS/MS-iTRAQ). The obtained data were analyzed using ProteinPilot software, classified by the Panther database and validated by western blots.

Hp, pCx and Cb tissues were isolated from four AD females and four age-matched non-demented females (controls) using the above-mentioned protocol. Detailed diagnostic features used in this study, such as stages of plaques and NFTs of AD patients and control patients, are described in Table 1. Proteomic analysis was conducted in two batches (B-I and B-II). For each batch, we used four (four biological replicates) different Hp, pCx and Cb tissues pooled from four AD and four control patients, respectively. Each batch was then repeated six times to ensure high confidence in the detection of brain region-specific proteins regulated by aging-related neurodegeneration. The quality of the data set and instrumental reproducibility was achieved by comparing and combining three technical replicates after the samples were labeled with 113–118 isobaric tags and processed in 2D-LC-MS/MS (Figure 1).

To verify the tissue specificity and that the protein samples were covering the whole human brain tissue proteome, all the identified proteins were uploaded into JVirGel (http://www.jvirgel.de/), a database that creates a virtual 2D-gel picture (Supplementary Figure S1).^35^

In addition, we used online databases (for example, Panther (Protein Analysis Through Evolutionary Relationship) at www.pantherdb.org, UniProt, NCBI and ‘softberry' http://linux1.softberry.com/berry.phtml) to classify the functions of the iTRAQ-identified proteins modulated in the AD brains. Further biocomputational network analysis of the iTRAQ-identified proteins using the Ingenuity Pathways Analysis (IPA, http://www.ingenuity.com) offered us additional valuable clues about the complex interactions between these proteins in the AD brain.

Following the database search and classification of proteins, western blots were performed on randomly selected proteins to verify the iTRAQ values. The western blot images correlated well and thus validated the obtained iTRAQ values (Supplementary Figures S2 and S3).

### Statistical analysis

The data obtained by the western blot analyses are illustrated as the mean±s.d. Student's *t*-tests were used to determine statistical significance. SPSS 19.0 (Statistical Products and Service Solutions, IBM Corporation, Armonk, NY, USA) for Windows was used to perform an analysis of variance followed by Fisher's protected least significant difference *post hoc* tests, when warranted. For the iTRAQ analysis, ProteinPilot Software 3.0 (Applied Biosystems) was used. To be considered statistically significant, we required a probability value to be <0.05 (95% confidence limit, *P<0.05).^29, 32, 33^

## Results

### Identification of brain region-specific proteome changes in the Hp, pCx and Cb of AD

We identified a total of 950 proteins in the Hp, pCx and Cb through the use of iTRAQ. Of those identified proteins, 825 were quantified with a strict cutoff of an unused ProtScore ⩾2 as the qualification criteria. These results correspond to a peptide confidence level of 99% and an applied false discovery rate of 0.33% (<1.0%). A total of 31 proteins showed an altered expression level in the investigated brain sites (Figure 1 and Tables 2, 3, 4, 5, 6, 7, 8, with the cutoff for up- and down-regulation predefined at 1.2 and 0.83, respectively (*P*-values are the average *P*-values of batch-I and batch-II; s.d. values are s.d. of batch-I and batch-II)).

Specifically, 11 proteins (APP, MARCKS, INA, MECP, HIST1H1E, ALB, GNB1, AK1, ALDOA, TNR and CLU) in the Hp (Table 2), 2 proteins (SYN1 and ATP5A1) in the pCx (Table 3), and 7 proteins (PLP1, GAP43, DPYSL2, QDPR, MATR3, ENO1 and GSN) in the Cb (Table 4) were modulated. In addition, ANXA6 was modulated in both Hp and pCx (Table 5). MAPT, MAP1A, AHNAK, CEND1 and GAPDH were altered in both Hp and Cb (Table 6) and HIST1H1D and GLS were modulated in both pCx and Cb (Table 7). SOD2, MBP and VIM were modulated in all brain regions investigated (Table 8).

As expected, APP was found to be upregulated in the AD Hp, whereas MAPT and MAP1A were upregulated in both AD Hp and Cb. It is noteworthy that Clusterin (CLU) was downregulated in the Hp, AHNAK was upregulated in the Hp and downregulated in the Cb, the extracellular matrix protein Tenascin-R (TNR) was downregulated in the Hp, HIST1H1D/E were upregulated in the pCx and the Hp and downregulated in the Cb and MECP2 was upregulated in the Hp of AD brains. In addition, the antiapoptotic protein SOD2 was upregulated in all three areas (Hp, pCx and Cb) of AD subjects.

### Biocomputational classification of the regulated proteins in the Hp, pCx and Cb of AD brains

**Figure 2:**
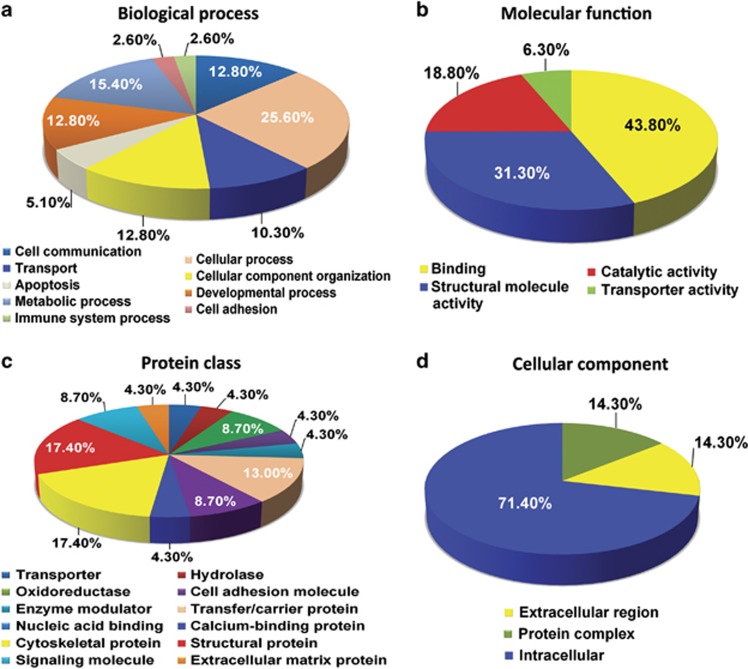
Pie chart depicting the functional classification of differentially regulated proteins in the hippocampus (Hp) of Alzheimer's disease (AD) brains. The isobaric tag for relative and absolute quantitation (iTRAQ)-identified hippocampal proteome was characterized within the molecular function Gene Ontology (GO) category. Subcellular and functional categories were based on the annotations of GO using the online tool at www.pantherdb.org in the following categories: (**a**) biological process, (**b**) molecular function, (**c**) protein class and (**d**) cellular component.

**Figure 3:**
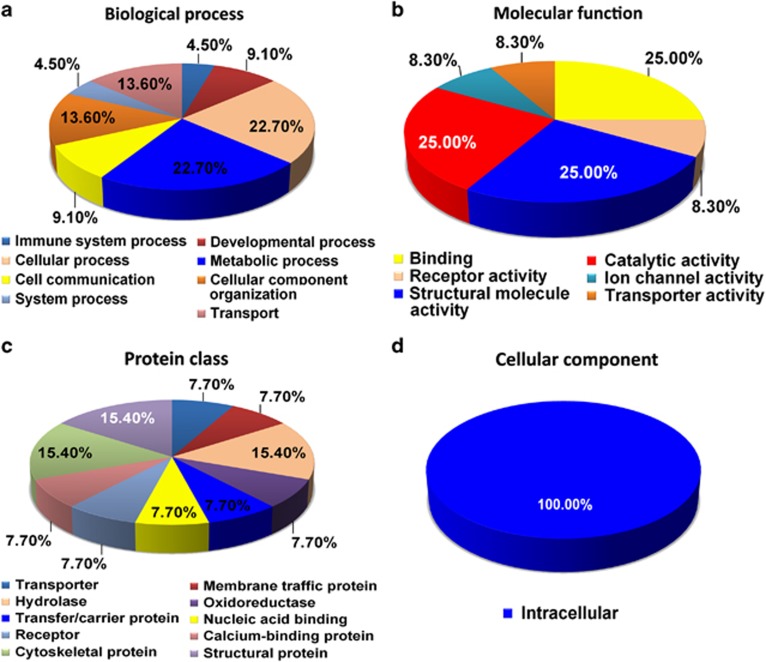
Pie chart depicting the functional classification of differentially regulated proteins in the parietal cortex (pCx) of Alzheimer's disease (AD) brains. The isobaric tag for relative and absolute quantitation (iTRAQ)-identified parietal cortical proteome was characterized within the molecular function Gene Ontology (GO) category. Subcellular and functional categories were based on the annotations of GO using the online tool at www.pantherdb.org in the following categories: (**a**) biological process, (**b**) molecular function, (**c**) protein class and (**d**) cellular component.

**Figure 4:**
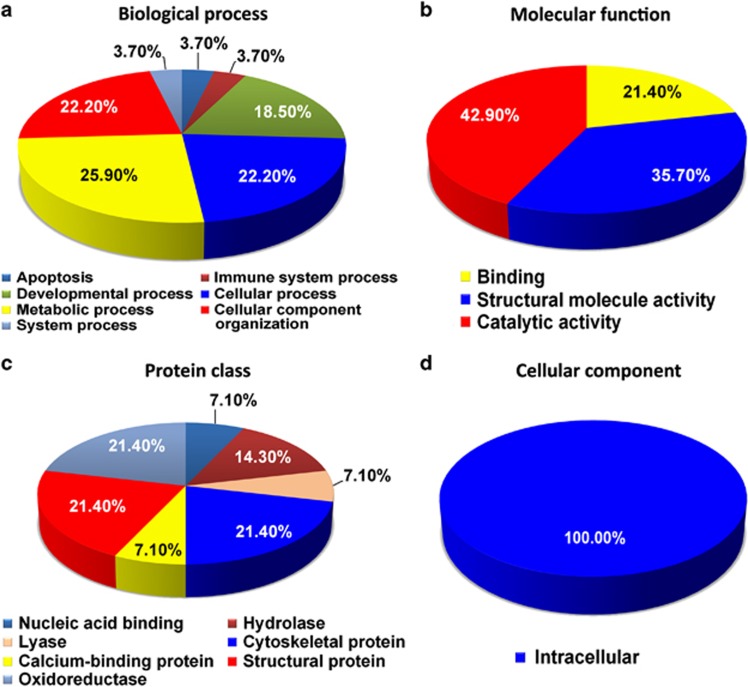
Pie chart depicting the functional classification of differentially regulated proteins in the cerebellum (Cb) of Alzheimer's disease (AD) brains. The isobaric tag for relative and absolute quantitation (iTRAQ)-identified cerebellar proteome was characterized within the molecular function Gene Ontology (GO) category. Subcellular and functional categories were based on the annotations of GO using the online tool at www.pantherdb.org in the following categories: (**a**) biological process, (**b**) molecular function, (**c**) protein class and (**d**) cellular component.

Next, we classified all of the 31 differentially regulated proteins from the Hp, pCx and Cb of AD brains based on their classes as well as cellular and molecular functions using the Panther database (Figures 2, 3, 4 and Tables 2, 3, 4, 5, 6, 7, 8). As predicted, both molecular and cellular functions of these proteins, as well as their associated biological pathways, were significantly altered in the Hp and the pCx compared with the Cb (Figures 2, 3, 4). All investigated regions (Hp, pCx and Cb) revealed severe alterations in metabolic, cellular and developmental processes. The most interesting observation was that the percentage of alterations in developmental processes was highest in the Cb (35.7% it was 29% for Hp and 25% for pCx), whereas the percentage of alterations in cellular processes was equivalent in both the pCx (62.5%) and the Hp (58%) (Figures 2, 3, 4a). As part of these changes, binding proteins (43.8% HIST1H1E and APP) were significantly altered in the Hp as well as the structural proteins (31.3% MAPT, MARCKS, MBP, INA and vimentin) and catalytic regulators (18.8% GAPDH and GNB1). Alterations in binding proteins (25% HIST1H1D and ANNEXIN A6), catalytic proteins (25% GLS, ATP5A1 and SOD2) and structural proteins (25% MBP, synapsin1 and vimentin) were similar in the pCx. Catalytic proteins (42.9% GLS, GAPDH, ENO1 and SOD2) in the Cb were the most affected group of proteins. Structural proteins (35.7% MAPT, GSN, PLP1, MBP and Vimentin) and binding proteins (21.4% HIST1H1D and GSN) were also significantly affected (Figures 2, 3, 4b). It is of interest to note that a substantial proportion of extracellular proteins (for example, TNR) were significantly downregulated exclusively in the Hp, whereas downregulation was only observed among intracellular proteins in the pCx and the Cb (Figures 2, 3, 4).

### Biocomputational network analysis of the proteins regulated in the Hp, pCx and Cb of AD brains

**Figure 5:**
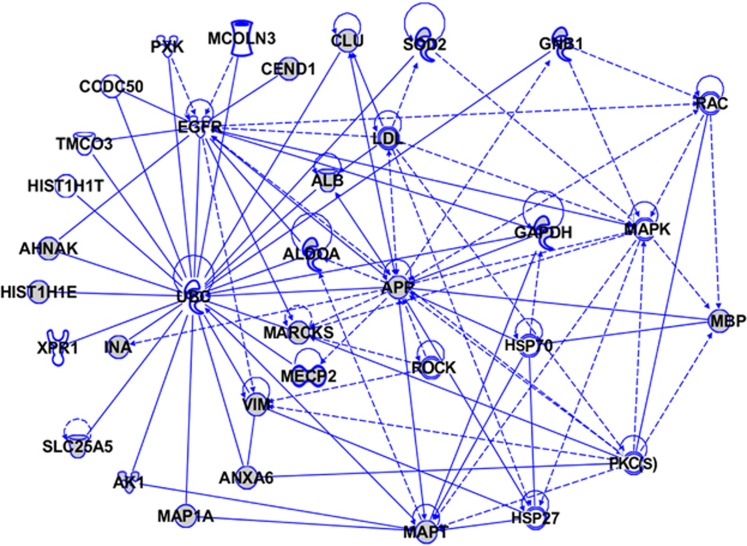
The Ingenuity Pathway Analysis (IPA)-generated network of isobaric tag for relative and absolute quantitation (iTRAQ)-identified proteins and their interacting partners in Alzheimer's disease (AD) hippocampus (Hp). The IPA construct networks based on the differentially regulated proteins and their potential link with other known proteins in human AD Hp. Solid and dashed connecting lines indicate the presence of direct and indirect interactions, respectively. Modulatory roles of proteins on the expression of other proteins are indicated by arrows.

**Figure 6:**
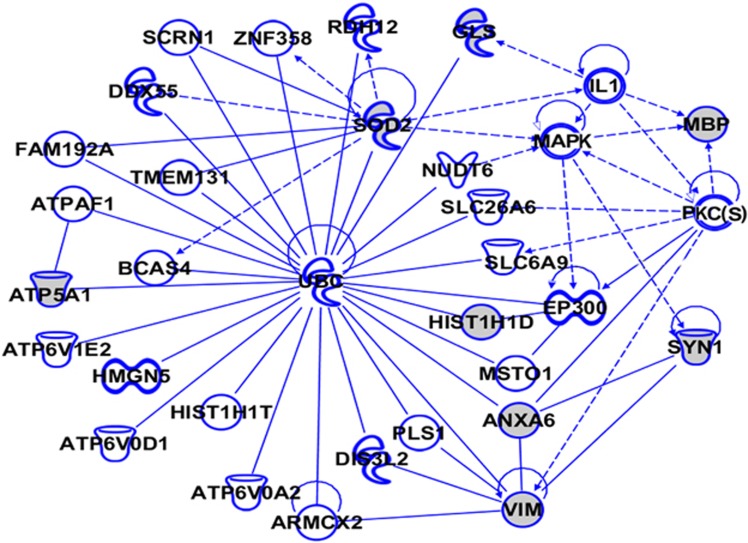
The Ingenuity Pathway Analysis (IPA)-generated network of isobaric tag for relative and absolute quantitation (iTRAQ)-identified proteins and their interacting partners in Alzheimer's disease (AD) parietal cortex (pCx). The IPA construct networks based on the differentially regulated proteins and their potential link with other known proteins in human AD pCx. Solid and dashed connecting lines indicate the presence of direct and indirect interactions, respectively. Modulatory roles of proteins on the expression of other proteins are indicated by arrows.

**Figure 7:**
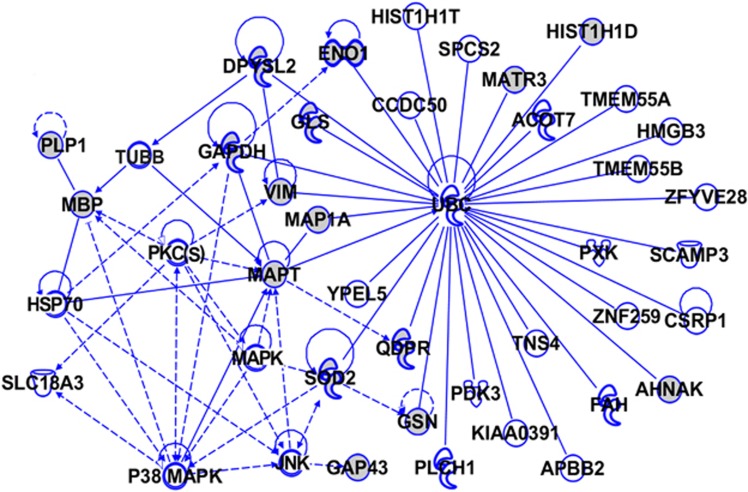
The Ingenuity Pathway Analysis (IPA)-generated network of isobaric tag for relative and absolute quantitation (iTRAQ)-identified proteins and their interacting partners in Alzheimer's disease (AD) cerebellum (Cb). The IPA construct networks based on the differentially regulated proteins and their potential link with other known proteins in human AD Cb. Solid and dashed connecting lines indicate the presence of direct and indirect interactions, respectively. Modulatory roles of proteins on the expression of other proteins are indicated by arrows.

To gain further insight into the potential biological mechanisms involving the iTRAQ-identified differentially regulated proteins in the AD brain, we used IPA (http://www.ingenuity.com). IPA identifies protein networks based on the known interactions (either direct or indirect) between proteins. In addition, IPA defines common functional and canonical pathways, thereby offering additional information about the complex interactive links between these proteins in the brain (Figures 5, 6, 7). The Hp network comprises 35 proteins (AHNAK, AK1, ALB, ALDOA, ANXA6, APP, CCDC50, CEND1, CLU, EGFR, GAPDH, GNB1, HIST1H1E, HIST1H1T, HSP27, HSP70, INA, LDL, MAP1A, MAPK, MAPT, MARCKS, MBP, MCOLN3, MECP2, PKC(s), PXK, RAC, ROCK, SLC25A5, SOD2, TMCO3, UBC, VIM and XPR1), of which 22 proteins were quantified by iTRAQ. The remaining 13 proteins were found by IPA to interact with the proteins quantified by iTRAQ. These proteins mediate biological processes such as glucose metabolism, signal transduction and apoptosis (Figure 5). The pCx network contained 33 proteins (ATP6V0D1, ATPAF1, ATP5A1, ATP6V1E2, ATP6V0A2, ANXA6, ARMCX2, BCAS4, DDX55, DIS3L2, EP300, FAM192A, GLS, HIST1H1D, HIST1H1T, HMGN5, IL1, MAPK, MBP, MSTO1, NUDT6, PKC(s), PLS1, RDH12, SYN1, SCRN1, SLC26A6, SLC6A9, SOD2, TMEM131, UBC, VIM and ZNF358), of which 8 proteins were quantified by iTRAQ. The remaining 25 proteins were found by IPA to interact with the proteins quantified by iTRAQ (Figure 6). The Cb network comprises 43 proteins (ACOT7, AHNAK, APBB1, CCDC50, CSRP1, DPYSL2, ENO1, FAH, GAPDH, GLS, GAP43, GSN, HIST1H1D, HIST1H1T, HMGB3, HSP70, JNK, KIAA0391, MAP1A, MAPK, MAPT, MATR3, MBP, P38MAPK, PDK3, PLCH1, PLP1, PKC(s), PXK, QDPR, SCAMP3, ZNF259, SLC18A3, SOD2, SPCS2, TUBB, TMEM55A, TMEM55B, TNS4, UBC, VIM, YPEL5 and ZFYVE28), of which 16 proteins were quantified by iTRAQ. The remaining 27 proteins were found by IPA to interact with the proteins quantified by iTRAQ. These proteins are mainly involved in developmental processes, and their downregulation is critically associated with various neurological, skeletal and muscular disorders (Figure 7). Interestingly, our IPA networks of differentially regulated proteins from Hp, pCx and Cb revealed a strong interaction between most of the AD-associated proteins (such as AHNAK, CLU, CEND1, SOD2, GNB1, ANXA6, ATP5A1, TNR and HIST1H1 variants) with ubiquitin C (UBC), a type of post-translational modification system. UBC binds to target proteins either as a monomer or as a polymer and forms a polyubiquitin chain to label those proteins for the ubiquitin proteasome degradation system (UPS).

## Discussion

Aging-related neurodegenerative disorders such as AD are multifactor disorders. These factors include a variety of brain changes that begin as many as 20 years before symptoms appear and involve the disruption of the most intricate neuronal networks regulating synaptic plasticity, signal transduction and transport. In this study, we provide for the first time a comprehensive quantitative brain site-specific proteome study of aging and aging-related AD-type dementia brains.

In recent years, several proteomic studies have described the differential protein expression profile in the brains of AD patients, with great emphasis on the cortical and hippocampal proteomes. Surprisingly, none of the reports focused on the cerebellar proteome despite its unique behavior of staying unaffected in AD. However, several reports have confirmed that the Cb also reveals molecular changes in response to AD, but this area of the brain likely counteracts those changes more efficiently.^17, 18, 19, 20, 21, 22, 23, 24^ Therefore, we aimed to compare the Cb proteome with the Hp and pCx proteomes to provide critical information on brain region-specific mechanisms that are counteracting the development and progression of aging and AD-related neurodegeneration and may illuminate how the Cb sequesters itself from neuronal death in AD. We identified 31 proteins that were altered significantly in the investigated brain areas, including Hp, pCx and Cb. The biocomputational protein classification analysis using the Panther database suggests that these proteins are involved in diverse biological processes but with different configurations in the Hp, pCx and Cb. The IPA indicates that the proteins that were altered in the AD brains had a strong interaction with UBC signaling in all three of the investigated brain regions. UBC mediates the polyubiquitination of proteins and consequently targets them to be transported to the proteasome for degradation back to the basic building blocks of cells so that they may be recycled elsewhere.

In addition, our data are in agreement with the expression levels of several proteins altered in the brains of AD-based neuropathology. For instance, ATP5A1 was suggested to contribute to neurodegeneration as it accumulates in the cytosol at early stages of NFT-based neurodegenerative processes.^36^ Furthermore, the protein levels of ALDOA were reported to be increased in AD hippocampal tissue.^37^ Our data are in line with previous studies of ATP5A1, which was downregulated in the Hp whereas ALDOA expression was upregulated.^38^ Impairment of brain metabolism has been recognized as a hallmark of AD, and the reduction of glucose utilization is paralleled by a decrease in the expression of glycolytic enzymes. We observed a significant downregulation of ALDOA in the Hp, ENO1 in the Cb and GAPDH in both the Hp and the Cb, thus explaining the impaired glucose metabolic system in AD.

Hyperphosphorylation of MAPT (tau) is closely related to various neurodegenerative diseases, including aging-related dementia such as AD.^39, 40^ For instance, deposition of hyperphosphorylated tau in the Cb of PS1 E280A AD has recently been reported.^18^ Many mechanisms could be involved in tau hyperphosphorylation, including the upregulation of tau kinases, the downregulation of phosphatases and other covalent modifications of tau.^7, 41, 42^ Although the MAPT hyperphosphorylation in the Hp and the pCx has been reported in neurodegenerative tauopathies,^43^ no thorough data have been reported yet regarding its changes in the Cb. Our data reveal high protein expression levels of MAPT in both AD Hp and AD Cb tissues. Recently, it has been hypothesized that a prion-like transmission of misfolded hyperphosphorylated MAPT or Aβ aggregates between neurons is one possible explanation for AD-associated anatomical irregularity and progression that appears in the absence of cortical Aβ pathology and MAPT lesions in the transentorhinal region. Misfolded MAPT in the neuronal cytoplasm may function as a seed that triggers hyperphosphorylation and misfolding of the natively unfolded MAPT protein. Disease progression is therefore associated with the intercellular transfer of pathogenic proteins, such as hyperphosphorylated MAPT aggregates.^44, 45, 46, 47^ This sheds further light on the current discussion that an impairment of the UPS is affected at early stages of AD,^48, 49^ and is in agreement with our current and previous data showing that pivotal proteins of the chaperone/proteasomal pathways are changed at early stages of AD.^14, 15^ It is possible that these abnormally high expressions of MAPT and the disturbed UPS could contribute to the hyperphosphorylation of MAPT in the Hp and eventually in the Cb too. In addition, Sepulveda-Falla *et al.*^18^ reported that the deposition of hyperphosphorylated MAPT in the Cb was found in AD and was caused by a presenilin-1 mutation E280A. Thus, combined with our proteomic results, the high expression of (hyperphosphorylated) MAPT in the Cb could also be involved in aging-related neuropathologies such as AD. However, this suggests that the Cb exploits cellular tools to inhibit hyperphosphorylated MAPT-based NFT formation. Furthermore, we identified several other proteins critically regulated in AD brains that could eventually become potential therapeutic targets for various neurodegenerative disorders. In the following sections, we describe some of these proteins with respect to AD.

### CLU and AD

CLU is another important protein involved in neurodegenerative diseases.^50^ Association of CLU gene polymorphisms with late-onset AD has been reported recently,^51, 52, 53, 54, 55^ and additional data provide a possible link between the CLU and APOE genotypes in the etiology of AD.^56, 57^ Some *in vitro* studies have demonstrated that at certain concentrations, purified CLU can interact with Aβ and result in an inhibition of fibril formation and thus functions as an extracellular chaperone that prevents the aggregation of nonnative proteins.^58, 59, 60^ In contrast, in a mouse model of AD, CLU was found to cause neuritic dystrophy by promoting Aβ plaque formation.^61^ This effect of CLU varied according to the amount of the prefibrillar substrate.^62^ Regardless, CLU could play an antiapoptotic function by interfering with the activity of the proapoptotic protein Bax.^63^ Overexpression of CLU has also been found in several human gland cancers.^64^ Thus, our data support the hypothesis that the strict downregulation of CLU in the AD Hp could contribute to neurodegenerative processes because of a reduced chaperone function and decreased antiapoptotic activity.

### TNR and AD

TNR is an extracellular glycoprotein known to be primarily involved in signal transduction and cell–matrix adhesion. Loss of TNR impairs cognition, synaptic plasticity and motor abilities in mice. Homozygous deletion of TNR is associated with intellectual disability and cognitive deficits.^65, 66^ Our data are consistent with these reports, indicating that TNR was significantly downregulated. However, this TNR downregulation only occurred in the Hp, and not in the pCx or Cb. Thus, we postulate that the downregulation of TNR expression might be one specific direct cause of impaired cognitive abilities in AD. Interfering with the TNR protein expression level could potentially be useful for novel AD treatment strategies especially through an improvement of Hp function.

### AHNAK and AD

Recent findings have disclosed the crucial role of AHNAK in myelination processes during development, neuronal plasticity and neuro-re-/de-generation events;^67, 68^ because the development of tau lesions in AD is traceable to differences between early- versus late-maturing oligodendrocytes and to the exceptionally protracted myelination of late-developing portions of the human brain,^69, 70, 71^ AHNAK becomes of pivotal interest for future investigations.

### Gelsolin (GSN) and AD

Previous studies have identified the antiamyloidogenic role of gelsolin in AD. Gelsolin can reduce the amyloid burden by acting as an inhibitor of Aβ fibrillization and as an antioxidant and antiapoptotic protein.^72, 73^ However, the expression level of GSN in the brain of AD patients has been discussed controversially.^74, 75^ Our data indicate that GSN is significantly downregulated in the AD Cb, which is consistent with a recent report that a reduced plasma GSN level in AD patients was found. This suggests that GSN may function as an additional plasma biomarker candidate that could contribute to the diagnosis of early-stage aging-related AD.^75^

### MECP2 and AD

Environmental factors, including metals and dietary factors, operate by interfering with the interaction of methylated CpG clusters and binding proteins, such as MeCP2 and SP1. The impact of these factors on AD has been discussed previously.^76^ MECP2 was found to be involved in various neurodevelopmental disorders^77^ and to promote neuronal death.^78^ Thus, the extremely high expression of MECP2 in the AD Hp could contribute to neurodegenerative pathways.

### Histones and AD

The post-translational modifications of histones, such as acetylation and deacetylation, have been increasingly recognized as critical factors affecting gene activation and silencing in the brain of individuals with neurodegenerative disorders.^79, 80, 81, 82, 83^ Aside from its post-translational modifications, the expression levels of histones are also important for cell cycle control.^84^ Here, we show that the expression of HIST1H1E was increased in the AD Hp, whereas the expression of HIST1H1D was increased in the AD pCx but decreased in the AD Cb. In general, increased levels of histone variants were found in affected areas (pCx and Hp) in AD, whereas the Cb reflected reduced levels of another cell cycle protein, CEND1. The different changes in expression levels of histones in different brain regions indicate their brain site-specific roles in the pathology of AD. However, further studies are required to unravel the link between the expression levels and cellular functions of these proteins to accurately evaluate their specific pathological roles in AD and other neurodegenerative disorders.

### CEND1 and AD

CEND1 is another neural stem cell-specific protein involved in cell cycle exit and neuronal differentiation.^85^ The correlation of neurodegeneration and deregulation of the cell cycle in AD have been frequently discussed over the past few years.^86, 87, 88, 89, 90, 91, 92^ Brain site-specific CEND1 expression changes (increased in the Hp and decreased in the Cb) may cause neurodegeneration by promoting abnormal cell cycle re-entry.^93^ Thus, increased CEND1 expression in the Hp could be responsible for a reduced regeneration capacity, whereas its downregulation in the Cb might be a survival strategy acquired by this area to induce dynamic regeneration. Thus, our study suggests a potential therapeutic usage of CEND1 in neurodegenerative disorders.

### UPS and AD

**Figure 8:**
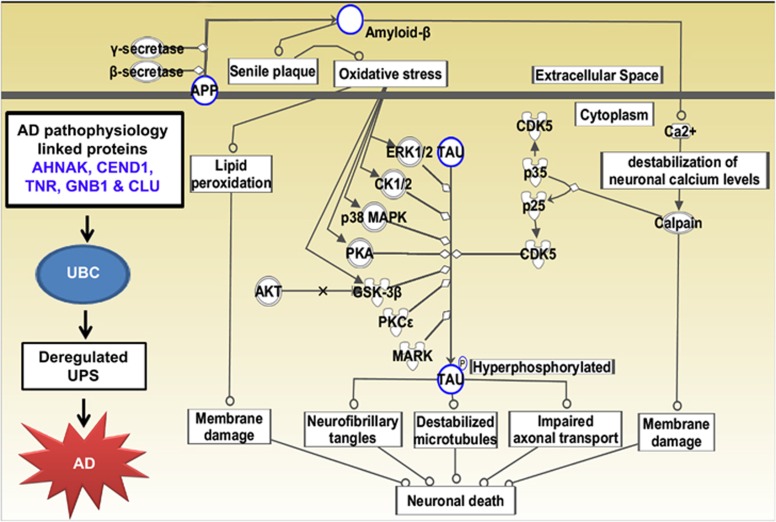
Ingenuity Pathway Analysis (IPA)-derived neurodegenerative disease-specific network of isobaric tag for relative and absolute quantitation (iTRAQ)-identified differentially regulated proteomes of hippocampus (Hp), parietal cortex (pCx) and cerebellum (Cb) of Alzheimer's disease (AD) brains. IPA analysis identified a group of proteins modulated in aging demented brain and their potential interactive links in the context of various neurodegenerative diseases. The nine different pathways identified by IPA are as follows: (1) amyloid processing (APP, MAPT), (2) Huntington's disease signaling (GNB1, GLS), (3) axonal guidance signaling (DPYSL2, GNB1), (4) mitochondrial dysfunction (SOD2, APP, ATP5A1), (5) glycolysis/gluconeogenesis (ENO1, ALDOA, GAPDH), (6) clathrin-mediated endocytosis signaling (CLU, ALB), (7) protein kinase A signaling (HIST1H1D, HIST1H1E), (8) signaling Rho family GTPases (VIM, GNB1) and (9) phospholipase C signaling (AHNAK, GNB, MARCKS).

Our iTRAQ data provide information about various proteins with an altered brain site-specific expression pattern, and it is intriguing to notice that many of them functionally interact with the pivotal UPS regardless of their resources. Our data are consistent with previous reports and reinforce that the function of the UPS is impaired in AD,^94, 95, 96, 97^ and thus emphasize the importance of the unfolded protein response and proteostasis in AD (Figure 8).^98^

The UPS is the major proteolytic pathway that degrades intracellular proteins in a regulated manner, usually essential for protein repair, turnover and degradation. Deregulation of the UPS has been implicated in the pathogenesis of many neurodegenerative disorders such as Parkinson's disease, Huntington disease, Pick's disease and prion diseases. In addition, deregulation of the UPS has also been implicated in several other genetic diseases, including cystic fibrosis, Angelman's syndrome and Liddle syndrome, suggesting that activation of similar mechanisms must occur in neurodegeneration as a basic phenomenon.^99, 100, 101, 102, 103^

Recently, it has also become clear that the UPS is perturbed in aging-related dementia such as AD.^96, 97, 104, 105, 106, 107, 108^ Pathological aging, with respect to protein repair and degradation via the UPS, and selective protein oxidation may cause protein damage, or protein mutations may induce a dysfunction of the proteasome and a deficit of the UPS in AD. Such events eventually lead to activation of cell death pathways and to an aberrant aggregation or incorporation of ubiquitinated proteins into hallmark structures of AD such as neuritic amyloid plaques or NFTs. In AD, insoluble Aβ peptide aggregates or inclusion bodies within nerve cells are commonly observed with ubiquitin immunoreactivity.^109, 110^ The accumulation of ubiquitin–protein conjugates in neuropathological lesions was first detected in NFTs isolated from human brain tissue,^111, 112, 113^ and it is these NFTs that best correlate with the degree of dementia.^13^ A more direct relationship between the ubiquitin system and pathogenesis of AD was established with the discovery of a frameshift mutation in the ubiquitin transcript, which results in an ubiquitin protein with 20 extra amino-acid residues at its C-terminus (UBB+1).^95, 108, 114^ However, it is not fully understood why ubiquitin is accumulated in intra- and extra-cellular deposits or how it is involved in AD pathogenesis. Our iTRAQ analysis provides compelling evidence that many of the altered molecular factors in AD brains interact with UBC. Thus, the interconnection of AD-related proteins with UBC implicates a pivotal role of ubiquitin UPS in aging-related neurodegeneration such as AD.

Although our proteomic data are in agreement with others,^115^ quantitative proteomics analyzing aging brain tissue has yet to be evaluated and reviewed with critical considerations. There are currently a combination of different methods used, such as (1) 2D difference gel electrophoresis (2D-DIGE) combined with MALDI-TOF-MS/MS (matrix-assisted laser desorption/ionization time-of-flight MS/MS) proteomics^17, 37, 38, 116^ or (2) 2D-LC-MS/MS-iTRAQ/ICAT (isotope-coded affinity tag); some proteins appear more frequently (for example, 14-3-3 proteins, VDACs, VIM, creatine kinase B, Aldolase, GFAP and HSP70 proteins), whereas others are hardly detected (for example, STAT3, p60TRP, NF-κB, NTRK1 and α-, β- and γ-secretases).^117, 118^ Despite improved sensitivity of our 2D-LC-MS/MS analyses, limitations of ‘undersampling' in LC-MS/MS still restrict the number of peptides that can be identified in a specific sample. Though this technical limitation can be assessed by the inclusion of technical replicates or identical samples that are processed in parallel,^119^ further technical improvements of quantitative 2D-DIGE/LC-MS/MS (iTRAQ/ICAT) proteomics are required to make more significant translational research-based contributions that may provide more clinically relevant data.

Further studies using higher numbers of control and AD patients, shorter post-mortem intervals and more individual patient-to-patient comparisons may certainly improve our knowledge regarding the neurodegenerative processes in the aging brain.

In conclusion, our current study demonstrates a brain site-specific aging-related proteome pattern and emphasizes the importance of UPS in the AD brain. We report higher MAPT expression levels in the AD Cb suggesting transmission of neurodegeneration signals to this area. Our study also indicates a crucial role of AHNAK in processes of aging-related dementia, and we hypothesize that the Cb exploits proteins such as CEND1 or GSN to fight against neurodegeneration. An even more comprehensive study of the cerebellar proteome would most likely shed further light on how this area counteracts NFT formation and AD-induced neurodegeneration.

In conclusion, our findings have unraveled the complex proteome changes that occur in aging brains and open a new avenue for drug discovery in aging-related brain disorders such as AD.

## Figures and Tables

**Table 1:** Characteristics of human brain subject tissue samples

*Subject (Patient)*	*Pathological diagnosis*	*Gender*	*Age (years)*	*Stage of amyloid deposits (none, A, B, C)*	*NFT stage (I–VI)*	*PMI (h)*
*Control*						
1	Physiological aging	F	82	A	I	34
2	Physiological aging	F	88	A	I	36
3	Physiological aging	F	87	A	II	7
4	Physiological aging	F	95	B	II	24
						
*AD*						
1	SDAT	F	80	B	VI	3.3
2	SDAT	F	88	C	V	11
3	SDAT	F	87	C	VI	6.5
4	SDAT	F	98	C	V	12

Abbreviations: AD, Alzheimer's disease; F, female; M, male; NFT, neurofibrillary tangle; PMI (h), post-mortem interval in hours; SDAT, senile dementia/Alzheimer's type. Stages of amyloid deposits: A, rare or a few; B, mild or moderate; C, numerous or marked.

**Table 2:** List of regulated proteins in the Alzheimer's disease (AD) hippocampus (Hp)

*Protein IDs*	*Gene symbols*	*Protein names*	*Biological process*	*No. of peptides*	*AD Hp/Control*	P*-value*	*s.d. value*	*Subcellular location*
*Structural constituent of cytoskeleton*								
IPI00219301.7	*MARCKS*	Myristoylated alanine-rich C-kinase substrate	Calcium-mediated signaling	30	2.05	4.43E–05	0.7725	Plasma membrane
IPI00001453.2	*INA*	α-Internexin	Cellular component morphogenesis	24	1.86	0.0349	0.1334	Intermediate filament
								
*Nucleic acid binding*								
IPI00645192.3	*MECP2*	Isoform B of methyl-CpG-binding protein 2	Regulation of transcription from RNA polymerase II promoter	11	3.35	0.0209	0.6794	Nucleus
IPI00217467.3	*HIST1H1E*	Histone H1.4	Nucleotide and nucleic acid metabolic process	12	7.65	0.0392	1.2124	Nucleus
								
*Transfer*								
IPI00745872.2	*ALB*	Isoform 1 of serum albumin	Transport	31	1.67	0.0155	0.3689	Secreted
								
*GTPase activity/ATP binding*								
IPI00026268.3	*GNB1*	Guanine nucleotide-binding protein G(I)/G(S)/G(T) subunit β-1	Ras protein signal transduction	6	0.71	0.0302	0.1165	Nucleus
IPI00640817.1	*AK1*	Adenylate kinase 1	ATP metabolic process	7	1.67	0.0307	0.2267	Cytoplasm
								
*Fructose binding/nervous system development*								
IPI00796333.1	*ALDOA*	Fructose-bisphosphate aldolase A	Glycolysis	26	0.57	0.0315	0.0111	Cytoplasm
IPI00160552.3	*TNR*	Tenascin-R	Signal transduction	10	0.67	0.0004	0.0238	Secreted
								
*Chaperone-mediated protein folding*								
IPI00400826.1	*CLU*	Isoform 2 of Clusterin	Apoptosis	3	0.51	0.0479	0.0168	Cytoplasm

**Table 3:** List of regulated proteins in the Alzheimer's disease (AD) parietal cortex (pCx)

*Protein IDs*	*Gene symbols*	*Protein names*	*Biological process*	*No. of peptides*	*AD pCx/Control*	P*-value*	*s.d. value*	*Subcellular location*
*Neurological system process/hydrolase activity*								
IPI00251507.2	*SYN1*	Isoform IB of Synapsin-1	Synaptic transmission	106	0.72	0.0334	0.0406	Golgi apparatus
IPI00440493.2	*ATP5A1*	ATP synthase subunit α	Cation transport	48	1.30	0.0424	0.0231	Mitochondrial

**Table 4:** List of regulated proteins in the Alzheimer's disease (AD) cerebellum (Cb)

*Protein IDs*	*Gene symbols*	*Protein names*	*Biological process*	*No. of peptides*	*AD Cb/Control*	P*-value*	*s.d. value*	*Subcellular location*
*Neurological system process*								
IPI00219661.2	*PLP1*	Isoform 1 of myelin proteolipid protein	Cellular component morphogenesis	6	5.77	0.0039	0.7291	Plasma membrane
IPI00015964.4	*GAP43*	Neuromodulin	Activation of protein kinase C activity by G-protein-coupled receptor protein signaling pathway	21	2.75	9.32E–05	0.1643	Cell membrane
								
*Hydrogen ion transporting ATP synthase activity/oxidoreductase activity*								
IPI00257508.4	*DPYSL2*	Dihydropyrimidinase-related protein 2	Nucleotide and nucleic acid metabolic process	47	1.69	0.0328	0.0542	Cytoplasm
IPI00014439.4	*QDPR*	Dihydropteridine reductase	Cellular amino acid catabolic process	11	1.59	0.0386	0.6406	Cytoplasm
								
*Nucleotide binding/lyase activity*								
IPI00789551.1	*MATR3*	Matrin-3	Structural molecule activity	6	0.66	0.0504	0.0304	Nucleus
IPI00465248.5	*ENO1*	α-Enolase	Glycolysis	42	0.75	0.0175	0.0980	Cytoplasm
								
*Structural constituent of cytoskeleton*								
IPI00026314.1	*GSN*	Isoform 1 of Gelsolin	Cellular component morphogenesis	10	0.69	0.0569	0.0540	Cytoplasm

**Table 5:** List of common regulated proteins in the Alzheimer's disease (AD) hippocampus (Hp) and parietal cortex (pCx)

*Protein IDs*	*Gene symbols*	*Protein names*	*Biological process*	*No. of peptides*	*AD pCx/Control*	P*-value*	*s.d. value*	*No. of peptides*	*AD Hp/Control*	P*-value*	*s.d. value*	*Subcellular location*
*Alzheimer disease-amyloid secretase pathway*												
IPI00219186.1	APP	APP Isoform APP714 of amyloid β A4 protein (Fragment)	Signal transduction	13	2.82	0.0128	0.0707	10	2.39	0.0136	0.2291	Membrane
												
*Calcium ion binding*												
IPI00221226.7	ANXA6	Annexin A6	Intracellular protein transport	17	1.83	0.0632	0.0487	18	2.64	0.0134	0.3616	Cytoplasm

**Table 6:** List of common regulated proteins in the Alzheimer's disease (AD) hippocampus (Hp) and cerebellum (Cb)

*Protein IDs*	*Gene symbols*	*Protein names*	*Biological process*	*No. of peptides*	*AD Hp/Control*	P*-value*	*s.d. value*	*No. of peptides*	*AD Cb/Control*	P*-value*	*s.d. value*	*Subcellular location*
*Structural molecule activity*												
IPI00220175.5	*MAPT*	Isoform Tau-E of Microtubule-associated protein tau	Cellular component morphogenesis	10	2.01	0.0013	0.2714	11	1.49	0.0371	0.0931	Plasma membrane
IPI00020356.4	*MAP1A*	MAP1A 331 kDa protein	Microtubule associated complex	12	1.51	0.0107	0.0183	17	1.70	0.0472	0.0222	Cytoplasm
												
*Protein binding*												
IPI00021812.2	AHNAK	Neuroblast differentiation-associated protein AHNAK	Nervous system development	8	2.73	4.84E–07	0.1066	6	0.55	0.0216	0.0239	Nucleus
IPI00295601.1	CEND1	Cell cycle exit and neuronal differentiation protein 1	Involved in neuroblastoma cell differentiation	6	5.7	0.0398	0.2243	2	0.52	0.0428	0.0205	Membrane
												
*Oxidoreductase activity*												
IPI00219018.7	GAPDH	Glyceraldehyde-3-phosphate dehydrogenase	Glycolysis	33	0.39	0.0027	0.0649	184	0.7955	2.12E–05	0.0621	Cytoplasm

**Table 7:** List of common regulated proteins in the Alzheimer's disease (AD) parietal cortex (pCx) and cerebellum (Cb)

*Protein IDs*	*Gene symbols*	*Protein names*	*Biological process*	*No. of peptides*	*AD pCx/Control*	P*-value*	*s.d. value*	*No. of peptides*	*AD Cb/Control*	P*-value*	*s.d. value*	*Subcellular location*
*DNA binding/hydrolase activity*												
IPI00217466.3	*HIST1H1D*	Histone H1.3	Nucleotide and nucleic acid metabolic process	7	4.12	0.0220	0.8771	13	0.57	0.0423	0.0852	Nucleus
IPI00289159.3	*GLS*	Isoform KGA of Glutaminase kidney isoform	Cellular amino acid metabolic process	3	0.35	0.0443	0.0229	14	0.81	0.059	0.0370	Cytoplasm

**Table 8:** List of common regulated proteins in the Alzheimer's disease (AD) parietal cortex (pCx), hippocampus (Hp) and the cerebellum (Cb)

*Protein IDs*	*Gene symbols/protein names*	*No. of peptides*	*AD pCx/Control*	P*-value*	*s.d. value*	*No. of peptides*	*AD Hp/Control*	P*-value*	*s.d. value*	*No. of peptides*	*AD Cb/Control*	P*-value*	*s.d. value*	*Subcellular location*
*Oxidoreductase activity*														
IPI00022314.1	*SOD2;* superoxide dismutase (Mn)	4	2.19	0.0406	0.6278	4	1.64	0.0393	0.2031	4	1.53	0.0452	0.1132	Mitochondrial
														
*Structural constituent of myelin sheath/structural constituent of cytoskeleton*														
IPI00021907.2	*MBP;* isoform 1 of myelin basic protein	61	2.05	0.0250	0.6187	30	1.53	9.31E–06	0.4068	61	3.40	2.6466E–05	0.4032	Cell membrane
IPI00418471.6	*VIM;* Vimentin	24	0.49	0.0039	0.0303	42	0.74	0.0241	0.0209	24	0.59	0.0213	0.0303	Cytoplasm
