# Supplementary material for: Brain site-specific proteome changes in aging-related dementia
Source: Exp Mol Med. 2013 Sep 6;45(9):e39–. doi: 10.1038/emm.2013.76 (PMC3789264; doi:10.1038/emm.2013.76)
Supplement: Supplementary Information [file emm201376x1.doc]

**Supplementary Data**

**- Materials and methods** *iTRAQ protocol*

**- Supplemental Fig. S1.** Simulated 2D-gel presentation of human AD brain-derived proteins quantified by iTRAQ.

**- Supplemental Fig. S2.** Western blot validation of randomly selected iTRAQ-quantified proteins significantly regulated in AD brains (parietal-cortex (pCx), hippocampus (Hp) and cerebellum (Cb)) compared with respective controls (Ctrl).

**- Supplemental Fig. S3.** Western blot validation of randomly selected iTRAQ-quantified proteins significantly regulated in AD brains (parietal cortex (pCx) only or hippocampus (Hp) only or cerebellum (Cb) only) compared with respective controls.

**Materials and methods**

*Antibodies*

Anti-ANXA6 (annexin A6, 1:5000, mouse monoloclonal, Sigma-Aldrich, WI, USA), anti-ACTB (beta actin, 1:3000, mouse monoclonal; Abcam, Cambridge, UK), anti-AK1 (adenylate kinase 1, 1:5000, mouse monoclonal; Abcam), anti-APP (1:10000, rabbit polyclonal; Sigma), anti-ATP5A1 (mitochondrial ATP synthase subunit alpha, 1:4000, rabbit polyclonal; Sigma-Aldrich), anti-CEND1 (cell cycle exit and neuronal differentiation 1, 1:10000, rabbit monoclonal; Abcam), anti-CLU (clusterin, 1:5000, mouse monoclonal; Upstate, Millipore, Merck, Billerica, MA, USA), anti-GAP43 (growth associated protein 43, 1:10000, rabbit monoclonal; Abcam), anti internexin (INA, 1:3000, rabbit polyclonal; Abcam), anti-MAPT (1:2000, goat polyclonal; Santa Cruz), anti-MBP (myelin basic protein, 1:500, rabbit polyclonal; Chemicon, Millipore, Merck), anti-QDPR (quinoid dihydropteridine reductase, 1:500, rabbit polyclonal; Abcam), anti-SOD2 (superoxide dismutase 2, 1:2000, mouse monoclonal; Abcam), anti-SYN1 (synapsin-1, 1:3000, rabbit polyclonal; EMD Millipore, Merck), anti-TNR (tenascin R, goat polyclonal; MyBioSource, San Diego, CA, USA), anti-VIM (vimentin, 1:4000, mouse monoclonal; Chemicon, Millipore, Merck).

*iTRAQ Protocol*

*Sample preparation - acetone precipitation*

Six hundred (600) μg of total protein lysates from each tissues were mixed with six volumes of 100 %, -20 °C-chilled acetone, vortexed thoroughly and incubated overnight at -20 °C for precipitation. The following day, samples were vortexed again and centrifuged at 16,000 x g for 30 min to pellet down all proteins. The supernatant was discarded and the pellets were washed in 500 μl of 90 %, -20 °C-chilled acetone. The washed pellets were allowed to air-dry at room temperature (RT) for 15 min, re-dissolved in 100 μl of 200 mM TEAB and 2 % SDS and then incubated at 50 °C for 5-10 min with gentle agitation using a thermomixer (Eppendorf). Following incubation the tubes were centrifuged at 16,000 x g for 30 min. The supernatant was collected and protein concentration was measured using the ‘2-D Quant’ kit (Amersham).

*SDS-PAGE and in-gel digestion*

Two hundred (200) μg of acetone-precipitated proteins (from each brain region) were mixed with protein loading dye, denatured for 10 min in a thermo bath (Fine PCR, Seoul, Korea) and resolved up to 60 % by SDS-PAGE. The gels were washed twice with autoclaved Milli-Q Water (MQW) for 5 min each and fixed overnight on a SH30L reciprocating shaker (Fine PCR) in 50 % methanol and 10 % Acetic Acid (AcOH). The gels were then washed with MQW thrice for 15 min each followed by in-gel digestion performed in a laminar flow hood (Gelman, Singapore). For in-gel digestion, the gels were diced into 1 – 2 mm pieces and transferred into tubes of 5 ml of 25 mM TEAB in 50 % Acetonitrile (ACN) buffer, vortexed and left at RT for 10 min to get rid of any impurity. After incubation buffer was discarded the washing step was repeated four times. Finally, 80 % ACN in 20 mM TEAB was added, vortexed and the tubes were left at RT for 10 min. The supernatant was discarded and the sample tubes were left to air-dry for 30 min.

*Reduction, alkylation, trypsin digestion and extraction*

Stock solutions of 200 mM tris (2-carboxyethyl) phosphine (TCEP) in HPLC water (J.T. Baker, Mallinckrodt, Inc., Phillipsburg, NJ, USA) and 200 mM S-methyl methanethiosulfonate (MMTS) in isopropanol were prepared. 5 mM of TCEP in 25 mM TEAB buffer was added to the dried gel pieces, vortexed and briefly spun before being incubated at 65 °C for 1 hr to allow reduction reaction to take place. Following this, 10 mM MMTS in 25 mM TEAB buffer (tube was covered with aluminum foil because MMTS is light sensitive) was added to gel pieces vortexed and briefly spun to allow alkylation reaction to proceed for 45 min at RT in the dark. The supernatant was removed and discarded. The gel pieces were again washed with 25 mM TEAB in 50 % ACN buffer as described above and then dehydrated by 100 % ACN. Finally, the tubes were air-dried for 30 min. Trypsinization was carried out in two steps; first step, 10 ml of 2.5 μg of trypsin in 25 mM TEAB buffer was added to each sample and incubated at 4 °C for 15 min for proper rehydration; second step, addition of further 10 ml of 2.5 μg trypsin solution in a 37 °C incubator overnight. Next day, the tubes were spun briefly and the aqueous extract of the digested proteins was collected. To the remaining gel pieces, 50 % ACN and 1 % AcOH was added, vortexed and incubated in a water bath sonicator for 30 min for the extraction of the peptides. The supernatant was transferred and combined to the main sample tube. The extraction step was repeated 5 times. The trypsin digested peptides of each samples were pooled and dried completely in a SpeedVac (Concentrator 5301, Eppendorf) at 30 °C and stored at -20 °C.

*Labeling of peptides with iTRAQ tags*

Each iTRAQ reagent tube content (tags- 113,114,115,116,117,118) was suspended in 70 μl of 100 % isopropanol and vortexed thoroughly. The dried peptides from each tissue samples were dissolved in 30 μl of 500 mM TEAB (dissolution buffer). Each iTRAQ tag was transferred to the respective peptide tubes and incubated at RT for 2 hr with gentle agitation (thermomixer). All individually labeled samples were then combined and dried in a SpeedVac at 30 °C.

*Desalting*

The dried peptide samples were reconstituted in 500 μl of 0.1 % formic acid (FA) and kept in the water bath sonicator for 5 min. Fifty mg C18 cartridge (Sep-Pak® Vac C18 cartridges, Waters, Milford, MA) was conditioned thrice with 100 % methanol pushed through at a rate of 2 to 3 drops per second via a syringe. The stationary phase was acidified three times with 0.1 % FA to condition the column. The sample was loaded into the column and allowed to flow via gravitational force and the flow-through was reloaded three times to avoid any loss of peptide. Next, the sample loaded column was desalted twice with 0.1 % FA and eluted using 75 % ACN + 0.1 % FA. This C18 desalting protocol was performed thrice with the desalting wash’s solution and the flow-through were combined. The samples were pooled, dried in SpeedVac, and stored at -20 °C.

*Electrostatic repulsion-hydrophilic interaction chromatography (ERLIC)*

Thousand two hundred (1200) µg of iTRAQ-labeled peptides were fractionated using PolyWAX LP weak anion-exchange column (4.6 × 200 mm, 5 μm, 300 Å; PolyLC, Columbia, MD, USA), within the Shimadzu HPLC system (Kyoto, Japan). The HPLC gradient used was composed of following buffers; 100 % solvent A (85 % ACN, 0.1 % AcOH, 10 mM ammonium acetate, 1 % FA, pH 3.5) for 5 min, 0 %–36 % solvent B (30 % ACN, 0.1 % FA, pH 3.0) for 15 min, 36 %–100 % solvent B for 25 min, and finally 100 % solvent B for 10 min. Gradient was allowed to run for 1 hr at a flow rate of 1.0 ml min^-1^. 29 fractions were collected after ERLIC which were reduced to 16 by pooling samples of similar peaks, as per the spectrum. The 16 sample tubes were dried in a SpeedVac and stored at -20 °C. The dried peptides were reconstituted in 100 μl 0.1 % FA for LC-MS/MS analysis.

*LC-MS/MS analysis­*

The samples were analyzed thrice (technical replicate = 3) for LC-MS/MS using a Q-Star Elite mass spectrometer (Applied Biosystems/MDS SCIEX) coupled with an online microflow HPLC system (Shimadzu). Multiple injections give a better coverage of the target proteome with superior statistical consistency. This is especially true for single peptide proteins as more MS/MS spectral evidence was obtained from multiple injections leading to higher confidence of peptide identification and quantification. The same pooled extracts were used for post-proteomics data validation using western blot analysis. Thirty μL of peptide mixture was injected and separated on a home-packed nanobored C18 column with a picofrit nanospray tip (75 μm i.d. × 15 cm, 5 μm particles) (New Objectives, Wubrun, MA, USA) for each analysis. The samples were separated at a constant flow rate of 30 μL/min with a splitter achieving an effective flow rate of 0.3 μL/min. Data acquisition was performed in the positive ion mode, with a selected mass range of 300-1600 m/z, and peptide ions with +2 to +4 charge states were subject to MS/MS. The three most abundant peptide ions above 5 count threshold were selected for MS/MS and each selected target ion was dynamically excluded for 30 s with 30 mDa mass tolerances. Automatic collision energy and automatic MS/MS accumulation were used to activate smart information-dependent acquisition (IDA). With maximum accumulation time being 2 s, the fragment intensity multiplier was set to 20. The relative abundance of the proteins in the samples was reflected by the peak areas of the iTRAQ reporter ions.

*Mass spectrometric data analysis*

The data was acquired with the Analyst QS 2.0 software (Applied Biosystems/MDS SCIEX). Using ProteinPilot Software 3.0, Revision Number: 114732 (Applied Biosystems), protein identification and quantification were performed. The peptides were identified by the Paragon algorithm in the ProteinPilot software and the differences between expressions of various isoforms were traced by Pro Group algorithm using isoform-specific quantification. The parameters used for the database search were defined as follows: (i) Sample Type: iTRAQ multi-plex (Peptide Labeled); (ii) Cysteine alkylation: MMTS; (iii) Digestion: Trypsin; (iv) Instrument: QSTAR Elite ESI; (v) Special factors: None; (vi) Species: None; (vii) Specify Processing: Quantitate; (viii) ID Focus: biological modifications, amino acid substitutions; (ix) Database: concatenated ‘target’ (International Protein Index (IPI) human; version 3.34; 69,164 sequences) and ‘decoy’ (the corresponding reverse sequences for false discovery rate (FDR) estimation); (x) Search effort: thorough. Pro Group algorithm was used to automatically select the peptide for iTRAQ quantification, where the reporter peak area, error factor (EF) and p value were calculated. Auto bias-correction was carried out on the acquired data to remove variations imparted as a result of unequal mixing during the combination of the differently labeled samples. PeptideProphet and ProteinProphet from Trans-Proteome Pipeline (TPP) were employed to estimate false discovery rates at both peptide and protein levels. Only protein groups with a probability above 0.9 were considered as identifications. To minimize the false positive identification of proteins, a strict cutoff of unused ProtScore ≥ 2 was used as the qualification criteria, which corresponds to a peptide conﬁdence level of 99 %. A FDR of 0.33 % (<1.0 %) was achieved. The cutoff for up- or down-regulation (pre-defined at 1.2 and 0.83 respectively) was determined by using the *p*-value cut-off of 0.05 to obtain the list of proteins with significant ratios. The *p*-value assigned by the ProteinPilot software measures the conﬁdence of the real change in the protein expression level. Then data analysis and functional classification were conducted using online databases (such as Panther (www.pantherdb.org), UniProt, NCBI, and ‘softberry’ (http://linux1.softberry.com/berry.phtml)).

*Post-proteomic data verification by SDS-PAGE and western blot analysis*

Twenty µg of cell lysates were resolved by 8-12 % SDS-PAGE at 0.02 Ampere (A) of constant current and transferred to a polyvinylidine fluoride (PVDF) membrane (0.22 μm; Amersham) using the ‘semi-dry’ transfer method (BioRad) for 60 min at 0.12 A in buffer containing 25 mM Tris, 192 mM glycine, 20 % methanol, and 0.01 % (wt/vol) SDS. The membrane was blocked with 5 % BSA (bovine serum albumin; BioRad) in Phosphate-buffered saline (PBS) plus 0.1 % Tween-20 (PBS-T) for 2 hrs at RT, washed three times in PBS-T for 10 min each, and incubated with primary antibody (diluted in 2 % BSA in PBS-T) for overnight at 4 °C. The membranes were washed as described above, incubated with HRP-conjugated secondary antibody for 1 hr at RT, and developed using the ECL plus western blot detection reagent (Amersham). X-ray films (Konica Minolta Inc., Tokyo, Japan) were exposed to the membranes before film development in a Kodak X-OMAT 2000 processor (Kodak, Ontario, Canada). For equal sample loading, protein quantification was performed using a ‘2D Quant’ kit (Amersham) with at least two independent replicates. BSA was used as a standard for protein quantification. To re-probe the same membrane with another primary antibody, Pierce’s (Pierce Biotechnology, Inc., Rockford, IL, USA) ‘stripping solution’ was used to strip the membranes. In addition, equal sample loading was confirmed using ACTB as a reference protein. Western blot experiments were performed at least three times for statistical quantification and analyses (n = 3), and representative blots are shown. Values (= relative protein expression) represent the ratio of densitometric scores (GS-800 Calibrated Densitometer and Quantity One quantification analysis software version 4.5.2; BioRad) for the respective western-blot products (mean ± SD (standard deviation)) using the ACTB bands as a reference for loading control.

*Virtual 2D-gel picture of the protein samples used*

In order to verify that the protein samples were indeed covering the whole brain tissue proteome, all the iTRAQ-identified proteins were uploaded into JVirGel (http://www.jvirgel.de/), a database widely used for the simulation and analysis of proteomics data. The virtual two-dimensional (2D) protein map shown in the supplemental figure 1 represents the distribution of the identified proteins according to their theoretical isoelectric points (pI) and calculated molecular weights (MW). Most of the identified proteins were having a molecular weight in the range of 10-420 kDa and distributed between an isoelectric point of 2.5 to 12.8. The proteome is a bimodal i.e. with two nodes, an acidic protein node and a basic protein node. Interestingly, the acidic node is comprised of more proteins with a closed distribution while the basic node is comprised of fewer proteins with a scattered protein distribution. The overall well dispersed protein spots in the virtual 2D gel image showed that the iTRAQ- identified proteins are from the whole brain cell proteome.

**Supplemental Figures**


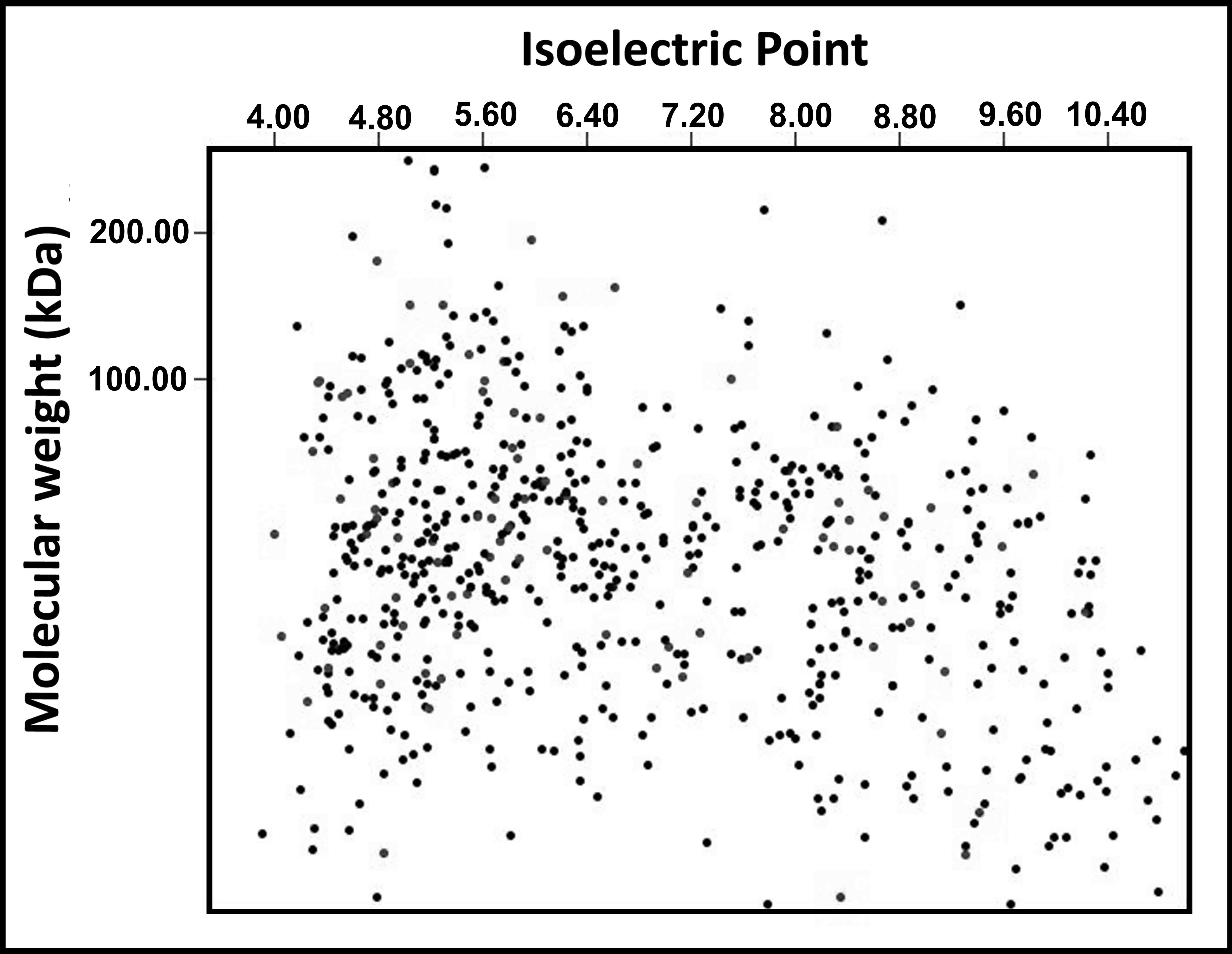


**Supplemental Fig. S1.** Simulated 2D-gel presentation of human AD brain-derived proteins quantified by iTRAQ. The proteins identified by LC-MS/MS were uploaded onto JvirGel, an online software used to create a virtual 2D-gel image. This image confirmed that the brain tissue-derived cell lysis was adequate and the entire proteome within cells was extracted. Isoelectric point and molecular weight values were generated from JVirGel at (<http://www.jvirgel.de/>).


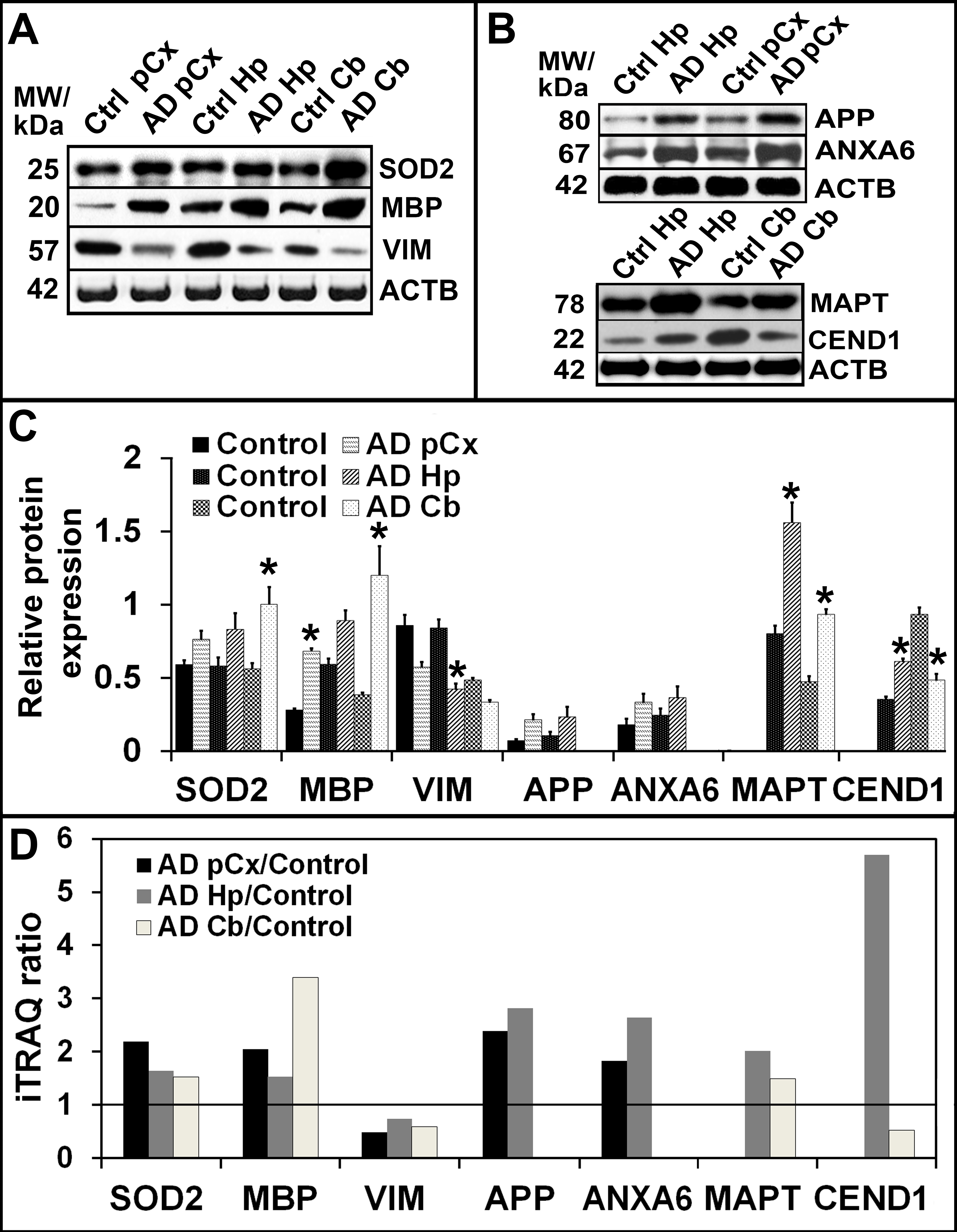


**Supplemental Fig. S2.** Western blot validation of randomly selected iTRAQ-quantified proteins significantly regulated in AD brains (parietal cortex (pCx), hippocampus (Hp) and cerebellum (Cb)) compared with respective controls (Ctrl). (A) Western blots against proteins SOD2, MBP and VIM that were modulated in all regions the Hp, pCx and Cb. (B) Western blots against proteins APP and ANXA6 that were modulated only in the Hp and pCx (upper panel) and against MAPT and CEND1 that were modulated only in the Hp and Cb (lower panel). In (A) and (B) ACTB was used as an internal control because its expression level remained unchanged in the iTRAQ analysis as well. (C) Quantitative analyses of the western blots shown in (A) and (B). Western blot experiments were performed at least three times for statistical quantification and analyses (n=3). Values (= relative protein expression) represent the ratio of densitometric scores for the respective western blot data and statistical error was indicated as mean ± SD (**P* < 0.05) using the ACTB bands as an internal control reference to ensure equal loading of the samples. The western blots correlated to the iTRAQ values obtained. (D) The histogram indicates similar ratios of the iTRAQ expression levels (AD vs controls) if compared with the western blot ratios for selected proteins (C). Thus, the western blot images correlated well and validated the iTRAQ values obtained.


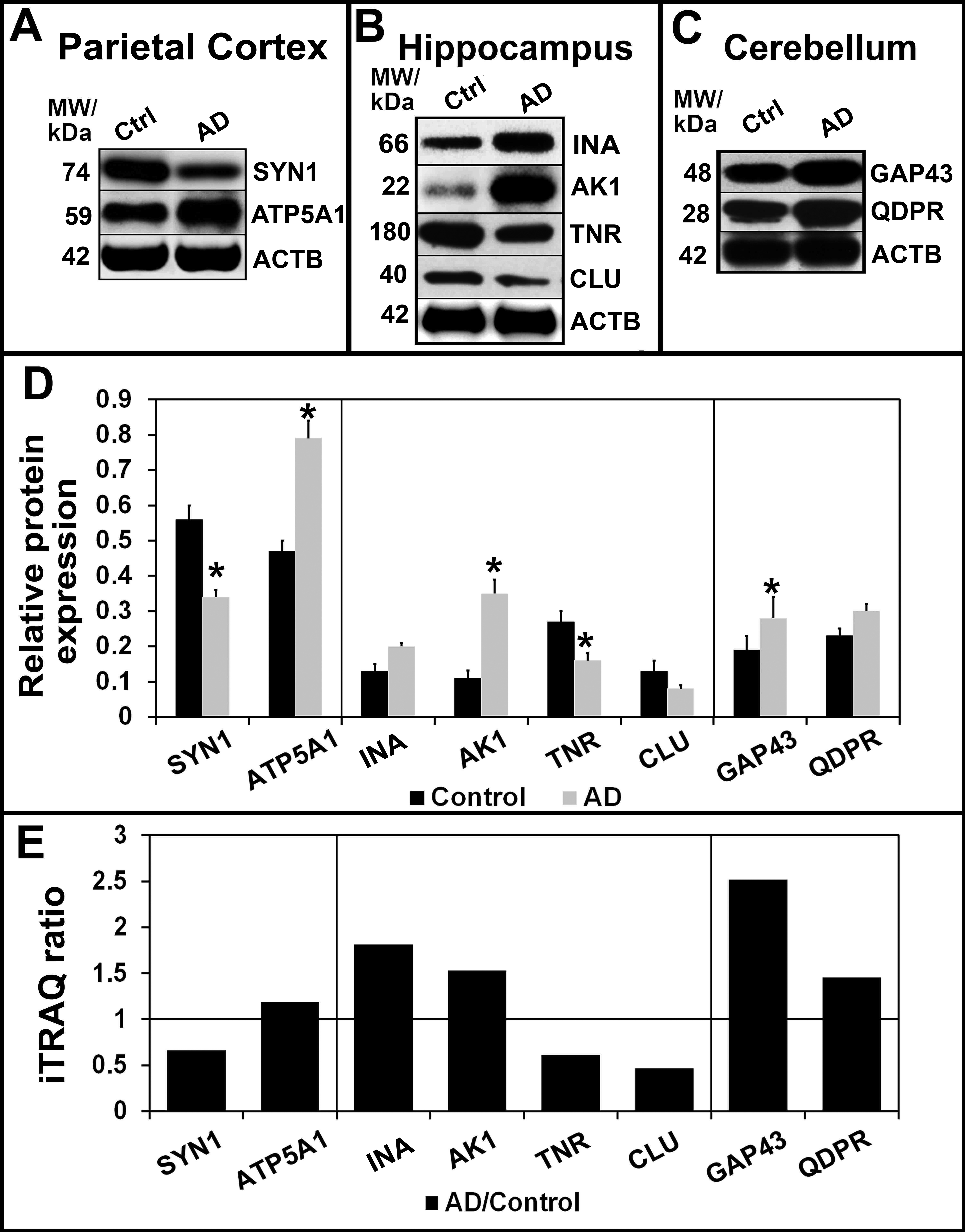


**Supplemental Fig. S3.** Western blot validation of randomly selected iTRAQ-quantified proteins significantly regulated in AD brains (parietal cortex (pCx), only or hippocampus (Hp), only or cerebellum (Cb) only) compared with respective controls (Ctrl). (A) Western blots against proteins SYN1, ATP5A1 that were modulated only in the parietal cortex of AD brain. (B) Western blots against proteins INA, AK1, TNR and CLU that were modulated only in the Hp. (C) Western blots against proteins GAP43 and QDPR) that were modulated only in the Cb. In (A), (B) and (C) ACTB was used as an internal control and because it remained unchanged in the iTRAQ analysis as well. (D) Quantitative analyses of the western blots shown in (A), (B) and (C). Western blot experiments were performed at least three times for statistical quantification and analyses (n=3). Values (= relative protein expression) represent the ratio of densitometric scores for the respective western blot data and statistical error was indicated as mean ± SD (**P* < 0.05) using the ACTB bands as an internal control reference to ensure equal loading of the samples. The western blots correlated to the iTRAQ values obtained. (E) The histogram indicates similar ratios of the iTRAQ expression levels (AD vs Ctrl) if compared with the western blot ratios for selected proteins (D). Thus, the western blot images correlated well and validated the iTRAQ values obtained.
